# Dementia Risk Reduction Education Programs and Resources for Indigenous Peoples of Canada, Aotearoa New Zealand, United States of America and Australia: A Scoping Review

**DOI:** 10.1177/14713012251355004

**Published:** 2025-06-26

**Authors:** Valda Wallace, Kathryn Meldrum, Yvonne Hornby-Turner, Rachel Quigley, Sarah Russell, Edward Strivens

**Affiliations:** 1 104560 College of Medicine and Dentistry, James Cook University, Cairns Campus, Cairns, QLD, Australia; 2 Education Design, Quality and Standards, 8001 James Cook University, Cairns Campus, Cairns, QLD, Australia; 3 Cairns and Hinterland Hospital and Health Service, 1288 Queensland Health, Cairns, QLD, Australia

**Keywords:** dementia risk, indigenous peoples, health promotion

## Abstract

Educational health promotion programs and resources support people to make informed decisions and change their behaviours. Dementia, a name for a group of degenerative brain diseases, affects over 55 million people across the globe. Currently, dementia risk reduction (DRR) is a global health priority as dementia has no known cure. Consequently, educational programs and resources that focus on DRR respond to the global health priority by targeting potentially modifiable risk factors. A project currently being undertaken by the research team is focussed on supporting DRR in Aboriginal and Torres Strait Islander Peoples’ primary care settings in Queensland, Australia. One strategy adopted by the research team is to identify safe and appropriate DRR programs and resources that could be integrated into primary care settings. Consequently, the aim of this scoping review was to identify and determine the quality of DRR programs or resources that have been developed or used with Indigenous peoples of Canada, Aotearoa New Zealand, the United States of America, and Australia. The Joanna Briggs method for scoping reviews was used to identify programs and resources developed with, for and by Indigenous peoples of the target countries. Appropriate databases including CINAHL and Medline as well as Google searches for grey literature published in English since 2010 were used to identify sources. Eleven sources were identified. One source was a published article, the other ten resources were videos (n = 5), websites (n = 2) and electronic written resources (n = 3). Given the paucity of evidence of DRR programs and resources currently available for Indigenous peoples the following recommendations are made for future development. They need to: (1). Be firmly grounded in Indigenous health promotion principles and theoretical frameworks and co-designed with, by and for Indigenous peoples. (2). Provide information about how dementia risk can be reduced; and (3). Linked with chronic disease interventions.

## Introduction

Education provided through health promotion programs and/or resources supports people to make informed decisions and act on their health behaviours and outcomes (Nutbeam & Muscat, 2021). Health promotion also has the potential to positively influence the “social, environmental and economic determinants of health” (Nutbeam & Muscat, 2021, p. 1580) of communities as well as individuals. The Ottawa Charter and World Health Organization proposed five health promotion action areas: (1) Policy; (2) the environment; (3) community action; (4) personal skills; and (5) health service reorientation (Nutbeam & Muscat, 2021; World Health Organisation, 2024). People can be supported to change their health behaviours by developing personal skills designed by health promotion programs, especially those that are focused on chronic disease risk factors (Francis & Baker, 2020). However, health promotion programs designed using Western orientated approaches may not be appropriate for Indigenous peoples (Dickerson et al., 2020; O’Keefe et al., 2022; Sacca et al., 2022; Walters et al., 2020). Consequently, cultural tailoring (Kreuter et al., 2003) or a more appropriate paradigm such as Indigenous health promotion (Durie, 2004; Harding et al., 2021; Mahoney & Fleming, 2020) should be used (The Indigenous Cognition and Aging Awareness Research Exchange, 2017).

### Indigenous Health Promotion

The concept of health for Indigenous peoples of Canada, Aotearoa New Zealand, the United States of America (USA), and Australia is inclusive of a “connectedness with ancestors, family, community, and lands, as well as storytelling, ceremony, spirituality, cultural identity and engagement, and self-determination” (O’Keefe et al., 2022, p. 2). This view is made clearer when exploring Indigenous Knowledge (IK) as the foundation of Indigenous peoples’ epistemology, ontology, axiology, and methodology. Although diverse worldwide, Indigenous peoples’ “IK has sustained their communities and includes a deep belief in the connectedness of all creation across time and space, with relationships between past, present, and future entities” (Walters et al., 2020, p. S56). Following the signing of the Alma-Ata Declaration and the move to primary healthcare, health promotion programs emerged to support and enhance this concept of holistic wellbeing (Mikhailovich et al., 2007). However, as the abundance of literature attesting to poor health outcomes for Indigenous peoples worldwide grows, it should be noted that health promotion must be compatible with Indigenous peoples’ values, beliefs, worldviews and aspirations for programs to be useful and effective (Durie, 2004). This approach in Australia is further supported by advice that there is a “need to focus on a positive policy agenda with an emphasis on community-controlled organisations staffed by Aboriginal and Torres Strait Islander people for Aboriginal and Torres Strait Islander Peoples, in order to develop and implement health promotion strategies” (Mahoney & Fleming, 2020, p. 89). Also noteworthy is that although the social determinants of health as defined by the World Health Organisation are all very applicable, Indigenous peoples’ good health and ill-health are also linked to rituals, spiritual beliefs, discrimination, land dispossession, social exclusion, legislations and policies (Mungabareena Aboriginal Corporation, 2008). Therefore, Indigenous health promotion programs must be developed with, by and for Indigenous peoples.

### Dementia Risk Reduction

Dementia, a name for a group of degenerative brain diseases, affects over 55 million people across the globe (Alzheimer’s Disease International, n.d; World Health Organization, 2017). Rates of dementia diagnoses are projected to increase as the global population ages. Currently dementia has no known cure so prevention, through dementia risk reduction educational programs and resources, have become a global public health priority (Walsh et al., 2023). Potential modifiable health behaviours such as physical inactivity, hypertension, brain injury, hearing loss, consumption of more than the recommended weekly units of alcohol, obesity, smoking, depression, diabetes, social isolation, exposure to air-borne pollution and more recently LDL cholesterol and untreated vision loss have been identified contributing to increased dementia risk in middle- (45–65 years) and later-life (>65 years) (Livingston et al., 2024). As more research is being conducted other potential risk factors such as diet type (Agarwal et al., 2023), poor kidney function (Russell et al., 2022; Thompson et al., 2023) and trauma during childhood (Radford et al., 2017) have been identified. Consequently, linking DRR education programs and/or resources with those designed to support healthy behaviour choices for chronic diseases such as diabetes, kidney failure, heart disease and smoking could be a worthwhile approach (World Health Organization, 2021).

### Context of Scoping Review

Canada, Aotearoa New Zealand, the United States of America (USA) and Australia were chosen for this review, as they all share a common history of being colonised by the British (Cavanagh & Veracini, 2017). The literature describes recurrent behaviours associated with colonisation, notably Paradies (2016) who reported the impact on the world’s First Nations Peoples included a “range of practices, predominantly historical: war, displacement, forced labour, removal of children, relocation, ecological destruction, massacres, genocide, slavery, (un)intentional spread of deadly diseases, banning of Indigenous languages, regulation of marriage, assimilation and eradication of social, cultural and spiritual practices” (p. 83–84).

Despite the continuing impacts of colonisation, the number of older Australian Aboriginal and Torres Strait Islander Peoples is increasing, and the trend is projected to continue (Australian Institute of Health & Welfare, 2023). In addition, the Indigenous Peoples of Canada, Aotearoa New Zealand, the USA and Australia are all diagnosed with age-related health conditions, including dementia, earlier than the non-Indigenous populations of their respective countries (Dudley et al., 2019; Flicker & Holdsworth, 2014; Jacklin et al., 2013; Lewis et al., 2021). Many of the age-related health conditions have been linked with the ongoing impacts of colonialism, social disadvantage and loss of traditional lifestyles (Thompson et al., 2023). Dementia diagnoses may also occur earlier in Indigenous populations due to links with chronic diseases such as diabetes, heart and kidney disease across the life span (World Health Organization, 2021). A recent systematic review focussed on dementia prevalence amongst Indigenous populations of countries with a very high Human Development Index (Clarke et al., 2024) identified that Australian Aboriginal and Torres Strait Islanders have the highest age-standardised dementia prevalence ratios (2.5–5.2). Lower age-standardised rations were observed for Māori Peoples (1.2–2.0). Additionally, crude prevalence ratios of Canadian First Nations Peoples (1.3) and American Indians and Alaskan Natives (1.0–3.2) indicated that there is a greater prevalence of dementia in the Indigenous populations of these countries when compared with non-Indigenous populations.

By upholding the tenet with, by and for Indigenous peoples the first and second authors (VW and KM) worked collaboratively on this scoping review. Each author identifies their standpoint in relation to the work and themselves below.

The first author (VW) identifies as an Australian Aboriginal woman from Gugu Badhun Country in north Queensland, the author also has ties to the Torres Strait Islander Peoples through family, friends, and work colleagues. Her work experience with Aboriginal and/or Torres Strait Islander Peoples spans over four decades in both Queensland and the Northern Territory working in health, policing, family support, education, and academia/research. Born in the 1950s, it is these lived experiences of growing up and growing old while living and working with Aboriginal and/or Torres Strait Islander Peoples that the author uses to examine what Aboriginal people value as important in the ageing process.

The second author (KM) is a non-Indigenous Australian with Scottish and Irish heritage, who emigrated from Scotland with her family as a young child. She has lived in Australia for most of her adult life. The last 12 years have been largely spent in Far North Queensland where she has developed strong personal and professional relationships with Australian Aboriginal and Torres Strait Islander Peoples. She has worked on several health projects with and for Australian Aboriginal and Torres Strait Islander Peoples. The first two authors have known each other for ten years and actively worked together for the last two years.

The context for this scoping review is a current project “Strengthening Primary Health Care Services to Prevent Dementia in Aboriginal and Torres Strait Islander Communities” that has been funded by a Boosting Dementia Research Grant through the Australian National Health and Medical Research Council (GNT1172054). The broader project aims to strengthen the quality of clinical care and health services that are focussed on dementia risk. Australian primary care centres are ideally placed to support DRR through proactive health promotion (Flicker & Holdsworth, 2014). One strategy intended to support the broader project’s aim is to identify relevant, safe and appropriate DRR educational programs and/or resources for Australian Aboriginal and Torres Strait Islander Peoples. Consequently, the aim of this scoping review was to map the existing evidence of appropriate DRR educational programs and/or resources for Indigenous peoples of Canada, Aotearoa New Zealand, USA and Australia. For the purposes of this scoping review, appropriate was defined as DRR educational programs and/or resources that have been designed with, by and for (Kjerland et al., 2024) the Indigenous peoples of the target country.

### Research Questions

The overarching research question that guided this scoping review was: What is the current evidence of programs and/or resources designed or modified for use with Indigenous peoples of Canada, Aotearoa New Zealand, USA or Australia that support DRR?

The associated sub-research questions were:

1. How many programs and/or resources designed or adapted for Indigenous peoples are available?
2. What are the key characteristics of the programs and/or resources?
3. What health promotion design approaches and strategies are being used?
4. What underlying theories, content, delivery, and timing approaches have been used to design the program and/or resource?
5. Have the programs and/or resources been designed with, for and by Indigenous peoples of the target population?
6. What is the quality of the programs or resources?
7. What is the evaluative evidence about the merit, value or worth of the programs or resources?

## Methods

This scoping review was conducted according to the nine-step method proposed by the Joanna Briggs Institute (JBI) (Peters, Godfrey, et al., 2020) and the previously published protocol (Meldrum et al., 2024). The Preferred Reporting Items for Systematic Reviews and Meta-analysis Extension for Scoping Review (PRISMA-ScR) guidelines (Peters, Marnie, et al., 2020) were used to report the findings of the scoping review in addition to the PRISMA checklist (Page et al., 2021) (Supplementary File 1).

### Changes after the Protocol Was Published

The only change to the previously published protocol was the removal of sub-research question 3 – To what extent do the programs/interventions or resources adhere to the World Health Organisation (WHO) DRR principles? Principle C “Evidence based practice for dementia risk reduction and care” (World Health Organization, 2017, p. 5) which identified person-centred, cost-effectiveness, sustainability, affordability, taking public health and cultural principles into account, was appropriate for this scoping review. However, no evaluations of any of the data sources were available. Additionally, insufficient information was available in the data sources to determine how and whether they addressed the WHO DRR principles. Consequently, it was not possible to determine whether the sources addressed WHO DRR Principle C.

### Eligibility Criteria

Experimental and quasi-experimental studies using qualitative, quantitative, or mixed methods designs were eligible for inclusion in this review. Grey literature that reported or contained any detail of DRR programs and/or resources were also eligible for inclusion. Any type of systematic review was excluded because of the focus on program design, which is not routinely reported in secondary sources, but their reference lists were scrutinised for possible sources.

In line with ScR method, the Participant, Concept, Context (PCC) framework (Peters, Godfrey, et al., 2020) guided inclusion criteria. Inclusion criteria was:

1. Any sources that described and/or reported preliminary results or outcome(s) of a program or resources designed or adapted to support DRR in Indigenous peoples of Canada, Aotearoa New Zealand, the USA or Australia.
2. Identified the approach used to design or develop the program and/or resource.
3. Used primary data.
4. Was a complete paper, report, resource or evaluation.

Sources were excluded from the data set if they were not explicitly and specifically focussed on DRR. For example, there are a significant number of dementia health literacy resources. These were excluded from the data set as they were focused on developing dementia health literacy, not DRR. Additionally, programs and/or resources addressing dementia risk factors (e.g. smoking) were not included because they did not specifically target DRR.

### Information Sources

The intent of this scoping review was to map the available evidence of DRR programs and/or resources designed or adapted with, for and by Indigenous peoples of Canada, Aotearoa New Zealand, the USA and Australia, published or unpublished studies and other grey literature sources, such as fact sheets, websites and audiovisual materials were located using the three-step strategy proposed by Peters, Marnie, et al. (2020).

CINAHL, Informit (Health and Indigenous Peoples Collection), Medline (Ovid) PsychInfo, PubMed and SCOPUS databases were searched Google, Google Scholar, and websites of dementia organisation websites in each country were also searched. Searches were completed between December 2023 and January 2024.

### Search Strategy

Published studies and other data sources written in the English language and published since January 2010 were sought for inclusion in the data set. English language sources were sought because it is the first language of the authors. Sources published since 2010 were sought because prior to that year there was little published literature on DRR and health promotion as a focus of DRR was made by the WHO in 2017. Consequently, few DRR programs and/or resources are likely to have been published before 2010. Search strings are available on Open Science Framework (OSF) (https://osf.io/u3mqf).

### Selection Process

All searches were carried out ‘by hand’ as the authors did not have access to automated tools such as Covidence. Initial database searches were performed by the second author (KM) and duplicates removed. Subsequently the first and second authors independently screened titles and abstracts (step two). The first and second authors then met to discuss the outcomes and resolve any differences in excluded and included sources. Step three, review of full-text records, weas also conducted independently. Grey literature sources were screened in the same manner. Endnote (Version 20.1) was used to manage sources across the selection process according to the method proposed by Peters (2017).

### Data Collection Process

An excel ™ database was designed by the second author to extract information from the data sources according to the PCC framework. Specific information related to the key characteristics of each of the sources was systematically extracted by the second author and subsequently checked by the first author. The database was collaboratively and iteratively refined during the data collection process.

In addition to the Excel spreadsheet for collating data from all sources, the TIDieR checklist (Hoffmann et al., 2014) was used to identify specific data contained in the single DRR intervention, the Dementia Prevention Program for Indigenous Australians (DAMPAA), in the data set (Mateo-Arriero et al., 2023). The purpose of the TIDieR checklist is to support authors to describe their intervention in sufficient detail to support replication. In this case, The TIDieR checklist enabled a deeper investigation of DAMPAA and extracted information that answered the scoping reviews research questions.

### Quality Appraisal

As the rationale for this scoping review was to identify and map existing evidence of DRR programs and/or resources for possible inclusion in a project currently being undertaken, quality appraisal was necessary. Two quality appraisal tools were used, the Public Health Ontario Meta-tool for Quality Appraisal for Public Health Evidence (MetaQAT) (Public Health Ontario, 2023; Rosella et al., 2016) and the Suitability Assessment of Materials (SAM) instrument (Doak et al., 1996). The MetaQAT was used to assess the quality of journal articles, as it focusses on assessing article quality from four perspectives: Relevancy; reliability; validity; and applicability.

The SAM (Doak et al., 1996) was used to determine the suitability of the written resources, which included websites and electronic resources housed on websites that were able to be printed out. The SAM is divided into six different sections: Appropriate content; literacy demand; graphics; layout and topography; learning and motivation; and cultural appropriateness. When considering the cultural appropriateness of the resources, the SAM section identifies a “match in logic, language and experience” and the use of “cultural images and examples” to assess cultural appropriateness. The format of the SAM enabled the authors, including the Australian Aboriginal author, to identify whether the resources included in the review met the SAM criteria.

### Data Synthesis

Qualitative summing and descriptive statistics that identified the key characteristics of programs and resources were used. A summary of the characteristics of DAMPAA from the TIDieR checklist. For the SAM, each written resource was scored against the criteria for each section. A score was calculated for each source and each section. Totals, percentages, and a rating was also attributed to each source. Content analysis (Kleinheksel et al., 2020) of all sources identified key characteristics, overarching health promotion design approaches, appropriateness of programs and resources for the target population and evaluative evidence of their efficacy. Key findings relevant to each research question are provided in tables with accompanying narrative that describes how the questions and findings are related.

## Results

This section presents a summary of the search strategy, the key characteristics of sources in the data set and then systematically discusses the key findings of the scoping review by addressing each research sub-question.

### Summary of Search Strategy

**Figure 1.**
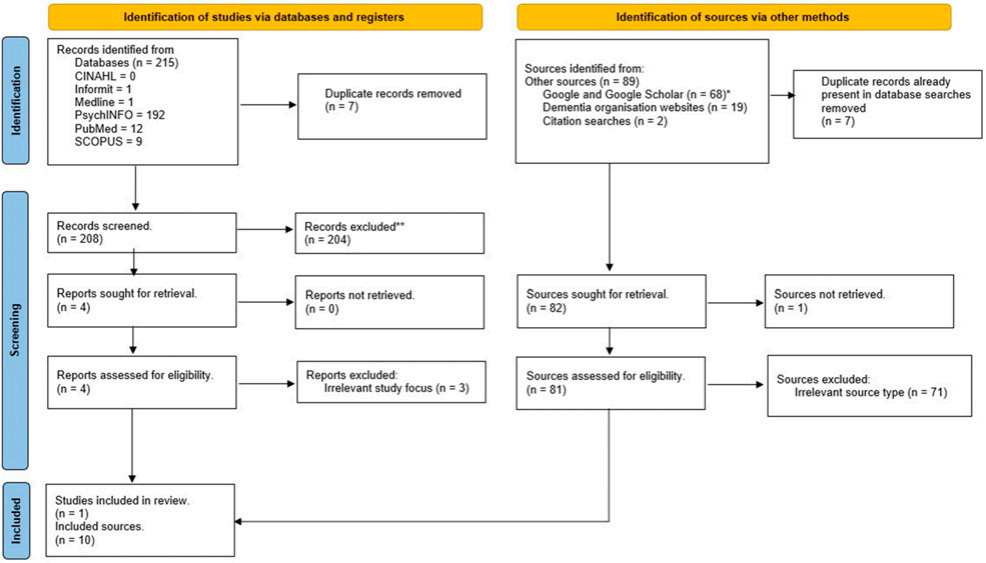
PRISMA Flow Diagram

Two hundred and fifteen (215) records were identified from database searches. After duplicates were removed (n = 7), 208 records were manually screened by the first two authors (VW and KM). Two hundred and four records (204) did not meet the eligibility criteria, hence four records remained for full screening. Only one of these records met the inclusion criteria for the scoping review. Grey literature searches including targeted searches of the websites of dementia organisations located in Canada, Aotearoa New Zealand the USA and Australia yielded 89 sources. However, Google and Google Scholar searches identified duplicates from the database searches that were subsequently removed (n = 7). One source was not able to be located from grey literature sources, which meant that 81 sources were reviewed. After all screening was completed, 11 sources constituted the data set (see Figure 1 PRISMA flow diagram). The accompanying data set is available from the OSF (https://osf.io/yh7w8).

### What is the Current Evidence of Programs and/or Resources Designed or Modified for Use with Indigenous Peoples of Canada, Aotearoa New Zealand, USA or Australia that Support DRR?

The scoping review identified three (27%) sources from Canada and eight (73%) sources from Australia. No Indigenous-specific DRR sources were able to be identified from Aotearoa New Zealand or the USA. Numerous dementia health literary sources from these countries were identified during screening. However, because their focus was not on DRR they were excluded.

Most sources were audiovisual (video) (n = 5; 45%). Three were electronic resources (n = 3; 27%) that were housed on websites and able to be printed out. Two websites (18%) housed information about DRR. The Anishinaabek Dementia Care website (https://anishinaabekdementiacare.ca/) focussed more on information specifically for that community. In contrast, the Neuroscience Research Australia (NeuRA) website (https://caringforspirit.neura.edu.au/research/reducing-risk-factors-and-preventing-dementia/) is inclusive of all Australian Aboriginal and Torres Strait Islander Peoples. Additionally, this website includes links to programs specifically focussed on dementia and chronic disease risk factors such as physical inactivity, smoking, drug use, and stress. Only one journal article (9%) that outlined the design and delivery of a DRR pilot program was included in the data set (Mateo-Arriero et al., 2023). Table 1 summarises the key characterises of the sources. Table 2 outlines the characteristics of the DAMPAA intervention (Mateo-Arriero et al., 2023) according to the TIDieR checklist.

**Table 1.** Summary Characteristics of Sources Included in the Scoping Review

Authors	Country of origin	Year	Resource type	Context	Population and sample size (n)	Key characteristics of program/intervention/resource
Anishinaabek Dementia Care (2024)	Canada	2024	Website	Community	n/a	Introductory web page that highlights four key points: Taking care of ourselves; taking time for each other; love; and, preparing for dementia
Consortium on Neurodegeration in Aging Team Advisory Group (n.d)	Canada	n.d	Electronic resource	Community	n/a	One page resource that overlays the medicine wheel with cultural activities and suggestions for DRR approaches
Dementia Australia (2022a)	Australia	2022	Video	Community	n/a	Aboriginal and Torres Strait Islander young people share key DRR approaches using hip-hop medium
Dementia Australia (2022b)	Australia	2022	Video	Community	n/a	Short video that focusses on maintaining brain health and looking after body; socialise, exercise; eat well. Avoid smoking, alcohol, and sugars. “Your brain matters” is the key message
Dementia Australia (2022c)	Australia	2022	Video	Community	n/a	Short video that focusses on maintaining culture and sharing stories, skills, and knowledge. An active brain, active body and strong heart facilitates maintaining and sharing culture
I-CAARE, (2017)	Canada	2017	Electronic resource	Community	n/a	Eight-page resource that outlines risk reduction approaches and includes quotes from carers
Mateo-Arriero et al. (2023)	Australia	2023	Journal article	Primary care	Aboriginal people 45 to 81 years (M = 63.5, SD = 11.7) n = 10	Outlines the plan for and delivery of a DRR pilot program called dementia prevention program for Indigenous Australians (DAMPAA). Education sessions × 6 planned (only 3 completed). Weekly exercise programming for one home exercise session per week. Group exercise mentioned but no details provided
Neuroscience Research Australia (NeuRA), (2019)	Australia	2019	Website	Community	n/a	Web page focusses on 4 key DR factors education, stress/trauma, head injuries and connection to community. Provides links to Aboriginal and Torres Strait Islander Peoples programs and/or resources for physical activity, smoking cessation, reducing head injury, drugs and alcohol and managing stress and wellbeing
The New England Dementia Partnership (2018)	Australia	2018	Electronic resource	Community	n/a	24-Page electronic resource is compiled as an e− book. Initially outlines what dementia is and then follows with 5 key DRR messages: Enjoy social life; exercise body; eat a healthy diet; take care of health (manage sleep problems; manage depression; avoid smoke and smoking; drink alcohol in moderation; and look after head)
University of Melbourne (n.d.)	Australia	n.d	Video	Community	n/a	Well known Aboriginal actor talks about the importance of brain health across the lifespan and provides strategies
University of Melbourne (2022)	Australia	2022	Video (webinar)	Primary care	n/a	Webinar focusses on DRR strategies

**Table 2.** TIDieR Checklist for Dementia Prevention Program for Indigenous Australians (DAMPAA) (Mateo-Arriero et al., 2023)

Brief name	Why		What
DAMPAA (Mateo-Arriero et al., 2023)	**Rationale:** Dementia risk is higher in Aboriginal Australians **Theory:** Theory of change **Goal:** Reduce cognitive decline and functional impairment in Aboriginal Australians 45 years of age and older (p. 4565)	**Materials:** Education session handouts, resources and worksheets, home exercise handouts and exercise calendar (p. 4567) *No indication of where materials can be accessed	**Procedures:** Initial assessment of 10 participants subsequently randomised into DAMPAA (n = 4) and control groups (n = 5) Control group completed usual routine (usual care).* DAMPAA was designed as a 12-month intervention with an action (1-6 months) and maintenance (7–12 months) periods **Action:** Two group exercise sessions per week and one home exercise session. Individual program goal setting completed in the first two weeks of commencement and reviewed every 4 weeks. Monthly education sessions delivered after the exercise session starting in week 5 of the program. Education focussed on exercise and brain health; good tucker (nutrition); diabetes prevention/management; medication management; falls prevention. Education program incorporated noongar Aboriginal language Pharmacist medication reviews Maintenance: Three home-based exercise sessions per week monitored by weekly phone check-in by DAMPAA staff Post 12-month intervention final assessment. (P. 4566–7; 4569) DAMPAA group only completed weeks 1–16 before pilot intervention ceased due to COVID-19 *Usual routine/usual care not described

Who	How	Where	When and how much
Aboriginal Health Practitioner (AHP) Exercise specialists.* *No information provided on expertise, background or specific intervention -related training provided	Face-to-face At home program	DAMPAA program delivered twice per week at aboriginal-controlled community health centre Derbarl Yerrigan	Face-to-face twice weekly and once a week at-home program.* *Program details intensity, duration and dose not described

Tailoring	Modifications		How well
Not applicable	Not applicable	**Planned:** Pilot was planned for twelve months. (P. 4569)*	**Actual:** Pilot intervention concluded at week 16 Only qualitative evidence from exit interview provided for efficacy of program. Participants reported exercises were appropriate (p. 4578)

### What Health Promotion Design Approaches and Strategies are Being Used?

The journal article authored by Mateo-Arriero et al. (2023) outlining the DAMPAA pilot was the only source where design approaches were able to be determined. The authors identified that Indigenous methodologies were used but not whether Indigenous health promotion principles underpinned DRR program design. Design approaches and strategies were not available for the other ten sources.

### What Underlying Theories, Content, Delivery, and Timing Approaches Have Been Used to Design the Program/Intervention or Resource?

Underlying health promotion theories were not able to be determined from Mateo-Arriero et al. (2023) journal article. However, the DRR program was co-designed and addressed education about chronic disease risk factors by providing opportunities for the participants to engage with exercise, nutrition, medication and falls prevention workshops. Due to COVID-19 only three workshops were completed. The DRR program was planned to be delivered face-to-face twice a week for 15 weeks. Participants also engaged with a planned individual exercise session at home during the week. A monthly exercise calendar and weekly exercise sheet with pictures and descriptions of each exercise were provided. Participants completed the weekly calendar and rated their perceived exercise exertion. Group physical activity opportunities are mentioned in the article, but no details are provided. Paper-based resources were provided to participants as workshop handouts and worksheets.

### Have the Programs and/or Resources Been Designed with, For and by Indigenous Peoples of the Target Population?

Most sources explicitly indicated that they were designed with, for and by Indigenous peoples of the target population. For example, the DAMPAA program (Mateo-Arriero et al., 2023) was explicitly co-designed with input from community Elders and staff from the Aboriginal-Controlled Community Health Organization that hosted the pilot intervention. For two sources, the “Let’s talk about brain health” (University of Melbourne, n.d) and “Health prevention and promotion for cognitive impairment and dementia in Aboriginal and Torres Strait Islander Peoples attending primary care” webinar this is less clear, although Indigenous people participated in both videos. As most sources were not published journal articles, there was limited evidence of how they were designed and to what extent Indigenous peoples were involved with them.

### What is the Quality of Programs and/or Resources?

The MetaQAT (Public Health Ontario, 2023) was used to determine the quality of evidence provided in Mateo-Arriero and colleagues’ article (2023). Full details of the quality appraisal are available in the data set (https://osf.io/yh7w8). A summary is presented here. The Mateo-Arriero et al. (2023) study was extremely relevant to this scoping review as it outlined the design and conduct of a DRR pilot program with a small group of Aboriginal Australians from Western Australia. However, a significant number of gaps in reporting the design and conduct of the pilot DRR program mean that it could not be replicated (reliability). For example, the frequency of education sessions, an absence of discussion about group sessions, and details about home exercise programs would have assisted in determining study reliability.

Major gaps in reporting the study findings also meant the authors were unable to make a judgement about validity. For example, the results of the pilot program were not conclusive. While Mateo-Arriero and colleagues’ (2023) indicated that the results would inform the full program, the study’s conclusions were more closely focussed on using theory of change models to support co-design rather than the outcomes of the DRR pilot program. Finally, Mateo-Arriero and colleagues (2023) article had limited applicability to the scoping review because there was insufficient detail about the DRR program to enable it to be used to support the research team’s current DRR project.

Each written resource was scored against the SAM (Doak et al., 1996) criteria for each section. Table 3 below presents the detailed assessment for each source. Collectively the sources were of superior quality.

**Table 3.** Suitability Assessment of Electronic Resources

Reference	AC	LD	G	LT	LM	CA	Total	N/As	Factoring N/As	Revised score	Percentage (%)	Rating
Anishinaabek Dementia Care (2024)	6	9	1	3	2	4	25	5	10	34	74	Superior
Consortium on Neurodegeration in Aging Team Advisory Group (n.d.)	6	3	2	1	2	2	16	6	12	32	50	Adequate
I-CAARE, (2017)	5	5	7	4	2	4	27	5	10	34	79	Superior
NeuRA (2019)	6	4	2	2	2	1	17	8	16	28	61	Adequate
The New England Dementia Partnership (2018)	4	4	12	5	4	4	33	4	8	36	92	Superior
Means	5	5	5	3	2	3	24	6	11	33	71	Superior

Key: Appropriate content – AC; literacy demand – LD; Graphics – G; Layout and Topography – LT; Learning and motivation – LM; Cultural appropriateness – CA. Number of not applicable – N/As.

### What is the Evaluative Evidence about the Merit, Value or Worth of the Programs or Resources?

No evaluative evidence of any of the sources was able to be located.

## Discussion

This scoping review found 11 sources that met the inclusion criteria. Most resources were audiovisual, which potentially suits how Indigenous peoples learn and are therefore appropriate (Kitchenham, 2017; Li et al., 2021).

The paucity of programs and/or resources was surprising. Compared with the number of dementia health literacy resources providing information about what is dementia, the number of programs and resources addressing DRR is lacking. This would indicate a time lag between building dementia literacy and the development of DRR programs and resources to build on this increased awareness.

### DRR Programs and Resources are Needed

There is clear evidence of a strong link between dementia and other chronic diseases (Hassen et al., 2022). Dementia risk can be managed by addressing potentially modifiable risk factors such as diabetes, heart disease, kidney disease etc. However, managing other chronic disease risks is difficult when people generally do not know **how** to initiate healthy life choices.

For example, in Australia, research has identified that “regularly, Aboriginal peoples are reported as having “poor diets” and the focus of much research and intervention reported in the peer-reviewed literature has been on what needs to be done to “fix” the “poor dietary intake” of Aboriginal peoples” (Wilson et al., 2020, p. 2). However, a study addressing food insecurity and barriers to healthy eating habits in two Australian Aboriginal communities reported “parents/caregivers may not have skills in how to budget, prepare for meals or to write up a weekly shopping list” (Sherriff et al., 2022, p. 10). Therefore, it is paramount clinical practitioners know their audience so that they can suggest **how** people can make changes to their lifestyle that will support healthy eating (Wilson et al., 2020).

While most of the sources in the data set referred to healthy eating, being physically active etc., only one of the data sources (Neuroscience Research Australia (NeuRA), 2019) explicitly linked to programs that could support Indigenous peoples to understand how to change their health behaviours.

### Indigenous Health Promotion Approaches and Principles

It is important that DRR programs and resources are underpinned by Indigenous health promotion approaches and principles. While it is acknowledged that the IK of Indigenous populations is different in each of the countries that were the focus of this scoping review, several consistent approaches have been identified by authors.

Firstly, community-based participatory approaches (Dickerson et al., 2020; Walters et al., 2020) such as co-design (Harding, 2022) need to be at the centre of DRR health promotion initiatives. Secondly, partnerships need to be established in the community (Dickerson et al., 2020; Mahoney & Fleming, 2020). As Mungabareena Aboriginal Corporation (2008) pointed out “We don’t want to be consulted; we want to be at the table.” (p. 8). Thirdly, the worldviews of the Indigenous peoples need to be embedded in the DRR program/resource design (Dickerson et al., 2020; Harding, 2022; Mahoney & Fleming, 2020; Walters et al., 2020). For example, Walters et al. (2020) from the perspective of American Indians, Alaskan Natives and Native Hawaiian Peoples, suggested that instructions supporting health promotion should be made using ancient stories and teachings where possible, that “relational restoration” (Walters et al., 2020, p. s56) and decolonising the way that Indigenous peoples think and talk about their bodies should be encouraged. For the Māori Peoples, principles of Kaupapa Māori, using Māori worldview and principles (Wilson et al., 2022) should be embedded (Harding, 2022) in any health promotion initiative.

### Findings Related to the Broader Study in which the Scoping Review is Situated

The intent of this scoping review was to identify DRR programs and/or resources designed with, by and for Indigenous peoples of Canada, Aotearoa New Zealand, USA and Australia that may be able to be used to support a project currently underway in Aboriginal and Torres Strait Islander peoples’ primary health care centres in Queensland, Australia. There was only one DRR program included in the scoping review data set. Reporting of the DAMPAA pilot study (Mateo-Arriero et al., 2023) provided limited details about the approach and outcomes of the program means that more information would need to be sought to replicate this program. Most sources in the data set are videos that could be incorporated into a bespoke resource or included in a DRR health promotion workshop conducted in the primary health care centres that are part of the project. The written resource developed by The New England Dementia Partnership (2018) has been used by the first author to support a DRR workshop and was well received by the participants. Each of the sources in the scoping review only provide parts of what is needed for a holistic DRR program that is appropriate for the communities involved in the project. This scoping review has enabled the project team to review what is needed to develop a holistic DRR program that is underpinned by Indigenous health promotion approaches and principles.

### Limitations

Limiting the data set to English language publications and resources may have decreased the number of sources included. There may be other sources in Indigenous peoples’ languages or French, for example, as Canada was included in the scoping review. Collaborating with researchers from the countries included in the scoping review may, therefore, have increased its reach.

## Conclusion

This scoping review mapped the extent of DRR programs and resources designed with, by and for Indigenous peoples of Canada, Aotearoa New Zealand, the USA and Australia. Results identified one published program and several other sources including videos, websites and written resources. Limited published DRR programs and/or resources specifically designed with, by and for Indigenous peoples of the target country are a concern. Indigenous-specific DRR programs and/or resources are urgently needed as health promotion has been recommended by the WHO as one of the key strategies for decreasing the rates of dementia worldwide (World Health Organization, 2017). However, DRR program and/or resource creators need to authentically partner with Indigenous peoples and use community-based participatory or co-design methods underpinned by Indigenous health promotion approaches and principles. Additionally, linking with existing chronic disease programs as well as supporting people to understand how to change their health behaviours could improve the efficacy of DRR programs and/or resources designed for Indigenous peoples.

## Supplemental Material

Supplemental Material - Dementia Risk Reduction Education Programs and Resources for Indigenous Peoples of Canada, Aotearoa New Zealand, United States of America and Australia: A Scoping Review Supplemental Material for Dementia Risk Reduction Education Programs and Resources for Indigenous Peoples of Canada, Aotearoa New Zealand, United States of America and Australia: A Scoping Review by Valda Wallace, Kathryn Meldrum, Yvonne Hornby-Turner, Rachel Quigley, Sarah Russell andEdward Strivens in Dementia
